# Case Report: Efficacy and safety of SGLT2 inhibitors in patients with Alström syndrome: a follow-up report of two siblings from the same family

**DOI:** 10.3389/fendo.2026.1785257

**Published:** 2026-07-30

**Authors:** Bingjie Xue, Kailu Li, Yu Guo, Yuting Wu, Huifang Peng, Liujun Fu, Hongwei Jiang

**Affiliations:** Luoyang Key Laboratory of Clinical Multiomics and Translational Medicine, Key Laboratory of Hereditary Rare Diseases of Health Commission of Henan Province, Henan Key Laboratory of Rare Diseases, Endocrinology and Metabolism Center, The First Affiliated Hospital, and College of Clinical Medicine of Henan University of Science and Technology, Luoyang, China

**Keywords:** ALMS1 gene, Alström syndrome, diabetes, ertugliflozin, insulin resistance, SGLT-2 inhibitor

## Abstract

**Background:**

Ertugliflozin, a sodium-glucose cotransporter-2 (SGLT2) inhibitor, is approved for the glycemic management of patients with type 2 diabetes. Alström syndrome (AS) is a rare autosomal recessive disorder caused by mutations in the ALMS1 gene, leading to ciliary dysfunction and multisystem involvement. AS is characterized by progressive neurosensory decline and metabolic disturbances, with symptoms such as retinal degeneration, which begins in childhood, sensorineural hearing loss, obesity, and cardiomyopathy. The 2020 European Alström Syndrome Clinical Management Guidelines, developed by experts from various medical fields, offer evidence-based recommendations for managing these symptoms and improving the quality of life of affected individuals. Severe insulin resistance, type 2 diabetes, non-alcoholic fatty liver disease, and renal failure significantly affect the quality of life and lifespan of patients with AS.

**Objective:**

To investigate the efficacy and safety of ertugliflozin in improving glycemic control, lipid metabolism, and insulin sensitivity in patients with AS.

**Methods:**

We retrospectively analyzed the clinical data of two sibling with AS treated at the Department of Endocrinology in our hospital. Follow-up data were collected and analyzed to evaluate the efficacy and safety of ertugliflozin in patients with AS.

**Results:**

During an 11-month observation period, ertugliflozin treatment exerted favorable effects on multiple metabolic parameters in the two patients. Glycemic control improved in both patients, as evidenced by significant reductions in HbA1c levels observed at 2 months. The homeostatic model assessment of insulin resistance (HOMA-IR) showed marked improvement in Patient 1. Patient 2 showed significant early improvement in HOMA-IR, but a rebound trend was observed later, potentially related to the inherent, rapidly progressive, severe insulin resistance characteristic of puberty in this patient. Further comprehensive evaluation is required to distinguish the long-term effects of SGLT2 inhibitors, such as ertugliflozin, from the progression of type 2 diabetes. Additionally, ertugliflozin demonstrated significant regulatory effects on lipid metabolism, and no adverse events occurred during the treatment period, indicating good safety.

**Conclusion:**

Consistent with current clinical management guidelines for Alström syndrome, ertugliflozin demonstrated clinically meaningful reductions in fasting and postprandial glucose concentrations in two patients with AS, as documented in this longitudinal case report. Modest improvements in total cholesterol levels were also observed; however, the isolated effect of ertugliflozin on triglycerides could not be ascertained in one patient due to concurrent fibrate therapy. Early enhancements in insulin sensitivity—assessed via HOMA-IR and dynamic glucose tolerance testing—were observed during the initial treatment phase; however, this effect waned over time in Patient 2, the younger patient. The agent was generally well tolerated, with no serious adverse events reported. Owing to the inherent limitations of this analysis—including a small sample size (n = 2), absence of a control group, and lack of histopathological or mechanistic biomarker data—these observations remain preliminary and warrant validation in adequately powered, prospective, controlled clinical trials.

## Introduction

Ertugliflozin (Steglatro) is a sodium-glucose cotransporter-2 (SGLT2) inhibitor that lowers blood glucose levels by inhibiting glucose reabsorption in the renal proximal tubules and promoting urinary glucose excretion. It is approved for glycemic management in patients with type 2 diabetes and has demonstrated cardiovascular and renal protective effects, particularly in adult patients with comorbid cardiovascular disease or chronic kidney disease.

Alström syndrome (AS), a rare genetic disorder characterized by mutations in the ALMS1 gene, results in ciliary dysfunction and is inherited in an autosomal recessive manner. It is associated with a range of symptoms, including vision impairment, hearing loss, obesity, and developmental delays, and currently lacks effective treatment. Clinical features involve multiple systems, primarily characterized by progressive neurosensory decline and metabolic disturbances. In infancy, patients may present with cone-rod dystrophy accompanied by nystagmus and severe visual impairment. During childhood, onset includes hearing loss, obesity, hyperinsulinemia, and severe insulin resistance. In late childhood and adolescence, diabetes, hypertriglyceridemia, dilated cardiomyopathy, and other multi-organ and metabolic abnormalities may occur (1). As the disease progresses, patients may develop hepatic, pulmonary, and renal dysfunction, along with multi-organ fibrosis, leading to reduced life expectancy.

Currently, there is no effective cure for AS, and management is primarily symptomatic, involving multidisciplinary interventions aimed at preventing and treating potential complications, alleviating symptoms, delaying disease progression, and improving quality of life and lifespan (2). Recent studies have demonstrated that SGLT2 inhibitors, including dapagliflozin and empagliflozin, are significantly effective in managing type 2 diabetes and improving cardiovascular outcomes, and these benefits may also extend to patients with AS (3). However, the use of ertugliflozin in patients with AS has not been reported. To our knowledge, this is the first report describing two patients with AS treated with ertugliflozin. In these patients, ertugliflozin was associated with significant improvements in glycemic control, lipid metabolism, insulin sensitivity, and insulin resistance, while demonstrating a favorable safety profile.

## Materials and methods

The investigational drug in this study, ertugliflozin, is a selective SGLT2 inhibitor. The pharmacokinetics of ertugliflozin are similar in healthy subjects and patients with type 2 diabetes. After once-daily administration of 5 mg, the steady-state mean plasma AUC and Cmax are 398 ng·hr/mL and 81.3 ng/mL, respectively. Steady state is achieved after 4–6 days of once-daily dosing. Following single oral administration under fasting conditions, peak plasma concentration is reached at approximately 1 h. The absolute oral bioavailability is approximately 100%. The mean elimination half-life is approximately 16.6 h. Plasma protein binding is 93.6%. Metabolism occurs primarily via O-glucuronidation mediated by UGT1A9 and UGT2B7, with CYP-mediated oxidative metabolism accounting for only approximately 12%. Regarding excretion, approximately 40.9% of the administered dose is excreted in feces and 50.2% in urine; 33.8% of the dose is excreted as unchanged drug in feces, and only 1.5% is excreted as unchanged drug in urine.

Currently, only metformin is approved for the treatment of type 2 diabetes in children and adolescents aged 10 years and older in China. Therefore, the use of ertugliflozin in this study constitutes off-label use. Considering that patients with Alström syndrome (AS) exhibit primary insulin resistance driven by ALMS1 mutations, and that SGLT2 inhibitors have an insulin-independent glucose-lowering mechanism, the clinical management guidelines for AS clearly recommend the use of the SGLT2 inhibitor empagliflozin for patients with comorbid chronic kidney disease. Previous case reports have also described the successful use of other SGLT2 inhibitors (e.g., dapagliflozin and empagliflozin) in patients with AS. After fully informing the patients’ guardians of the risks and potential benefits of off-label use, written informed consent was obtained, and ertugliflozin 5 mg once daily was added under close monitoring.

This study included two sibling patients diagnosed with AS, whose diagnoses were confirmed by genetic testing that identified ALMS1 gene mutations, and who had complete clinical data.

### Case 1

Patient 1, the elder brother, was diagnosed with Alström Syndrome (AS) at the age of 10 years through genetic testing. The testing identified compound heterozygous variants in the ALMS1 gene: c.1894C>T (p. Gln632*) and c.9148_9149delCT (p. Leu3050fs), which are indicative of the condition. The patient presented with symptoms consistent with Alström syndrome, including nystagmus, photophobia, amblyopia, and hearing loss, which were observed between the ages of 1 and 2 years and evaluated by specialists in the ophthalmology and ENT departments. Weight gain began at age 3. Acanthosis nigricans appeared on the neck and axillae at age 5 and remained untreated. Polydipsia, polyuria, and elevated blood glucose levels led to a diabetes diagnosis at an external hospital at age 8. At age 10, due to poor glycemic control and comorbid liver injury, the patient was hospitalized at another institution (HbA1c 9.18%), received hepatoprotective therapy, and started on insulin degludec (20 U/day, subcutaneously before breakfast) combined with metformin. Acarbose was added at age 11 due to suboptimal glycemic control.

At presentation to our hospital at age 16, physical examination showed short stature (height, 145 cm), weight 42 kg, waist circumference 70 cm, hip circumference 79 cm, acanthosis nigricans on the neck and axillae, vision limited to light perception, nystagmus, marked hearing impairment requiring the use of hearing aids, and no abnormalities on cardiopulmonary or abdominal examination. Laboratory tests indicated a significantly elevated HbA1c level of 10.9%, which suggests poor long-term glycemic control and a high risk of diabetes complications. Other clinical data are detailed in **Table 1**.

**Table 1:** Baseline and follow-up data of Patient 1.

Indicator	Baseline	After 2 months of treatment	After 5 months of treatment	After 11 months of treatment
Pathogenic variant	c.1894C>T (p.Gln632*) and c.9148_9149delCT (p.Leu3050fs)			
Age (years)	16			
Sex	Male			
Body mass index (BMI, kg/m²)	21.5	/	/	19.9
​Weight (kg)	44	/	/	42.2
Height (cm)​	143.1	/	/	145.5
​Fasting blood glucose (mg/dL)​	7.55	5.71	6.64	5.81
​Glycated hemoglobin (HbA1c, %)​	10.9	7.7	6.6	7.2
HOMA-IR	42.1	5.3	13.8	7.5
HOMA-β	619.8	189.3	298.7	250.3
Uric acid μmol/L	711	/	635	/
Creatinine (μmol/L)	83	/	/	112.9
eGFR ml/(min.1.73m²)	117.4	/	/	87.72
Total cholesterol	9.24	6.58	5.3	4.97
Triglycerides (TGs, mg/dL)	20.35	10.45	5.72	4.61
Low-density lipoprotein cholesterol (LDL-C, mg/dL)	0.79	/	/	1.98
High-density lipoprotein cholesterol (HDL-C, mg/dL)	0.55	/	/	0.79
Aspartate aminotransferase (AST, U/L)	29	21	22	33
Alanine aminotransferase (ALT, U/L)	20	25	31	67
Platelet count	159	/	/	212
APRI score	0.5	/	/	0.4
NAFLD fibrosis score (FIB-4)	1.84	/	/	0.58
Oral antidiabetic drugs (OADs)	Acarbose 50mg three times daily, metformin 0.5g twice daily	Acarbose 50mg three times daily, metformin 0.5g twice daily, ertugliflozin 5mg once daily	Acarbose 50mg three times daily, metformin 0.5g twice daily, ertugliflozin 5mg once daily	Acarbose 50mg three times daily, metformin 0.5g twice daily, ertugliflozin 5mg once daily
Lipid-lowering drugs	Bezafibrate dispersible tablets, 200 mg, three times daily	Bezafibrate dispersible tablets, 200 mg, three times daily	Bezafibrate dispersible tablets, 200 mg, three times daily	Bezafibrate dispersible tablets, 200 mg, three times daily

Baseline medications before adding ertugliflozin included acarbose 50 mg three times daily, metformin 0.5 g twice daily, and insulin degludec 20 U/day administered subcutaneously before breakfast. Due to persistent poor glycemic control, ertugliflozin 5 mg once daily was added. Additionally, for the treatment of comorbid hypertriglyceridemia (4), bezafibrate dispersible tablets 200 mg three times daily were administered. Glycemic control subsequently stabilized, and insulin therapy was gradually reduced and discontinued after one month (**Table 1**).

### Case 2

Patient 2, the younger brother, was born at full term without any abnormalities. However, visual abnormalities, including high myopia, decreased vision, photophobia, and nystagmus, were observed at the age of 5 months. These visual symptoms persisted for 1 year. Genetic testing at age 3 confirmed the presence of compound heterozygous ALMS1 variants, c.1894C>T (p.Gln632*) and c.9148_9149delCT (p.Leu3050fs), which were the same variants found in Patient 1, thus confirming the diagnosis of Alström Syndrome (AS). Progressive weight gain was observed during childhood. At the age of 6, the HbA1c level was 5.2%, indicating normal blood glucose levels. The insulin level was measured at 49.15 μU/mL, which was above the normal range, indicating insulin resistance. Consequently, metformin therapy was initiated. Fatty liver was detected at age 7; liver elasticity was 7.3 kPa (normal ≤7 kPa), suggesting signs of liver fibrosis. At the age of 8 years and 6 months, the patient’s fasting blood glucose was found to be 7.2 mmol/L, with an HbA1c of 7.5%. Given these levels, acarbose was introduced to complement the existing metformin therapy. The patient had repeated hospital visits because of abnormal liver function and intermittently received glucuronolactone.

At the age of 8 years and 8 months, he was hospitalized at our institution for abnormal liver function. Physical examination revealed obesity (height, 150 cm; waist circumference, 82 cm; hip circumference, 89 cm; weight, 54 kg; body mass index, 24 kg/m²), decreased vision, nystagmus, hearing loss, and axillary and neck acanthosis nigricans. No abnormalities were found on cardiopulmonary or abdominal examination. Laboratory tests showed HbA1c 9.9%. The other data are presented in **Table 2**.

**Table 2:** Baseline and follow-up data of Patient 2.

Indicator	Baseline	After 2 months of treatment	After 5 months of treatment	After 11 months of treatment
Pathogenic variant(ALMS1)	c.1894C>T (p.Gln632*) and c.9148_9149delCT (p.Leu3050fs)			
Age (years)	9			
Sex	Male			
Body mass index (BMI, kg/m²)	24	/	/	23.08
​Weight (kg)	54	/	/	55.1
​​Height (cm)​	150	/	/	154.5
Fasting blood glucose (mg/dL)	6.05	5.38	5.84	5.66
​Glycated hemoglobin (HbA1c, %)​	9.9	7.1	6.2	7.1
HOMA-IR	10	3.6	19.2	18.7
HOMA-β	291.4	161.2	632.7	688.7
Uric acid μmol/L	473	/	/	/
Creatinine (μmol/L)	29	/	/	27.9
eGFR ml/(min.1.73m²)	251.48	/	/	269.24
Total cholesterol	4.5	3.72	4.55	4.22
Triglycerides (TGs, mg/dL)	1.94	1.36	2.53	2.61
Low-density lipoprotein cholesterol (LDL-C, mg/dL)	2.87	/	/	2.56
High-density lipoprotein cholesterol (HDL-C, mg/dL)	0.96	/	/	1.03
Aspartate aminotransferase (AST, U/L)	260	51	39	86
Alanine aminotransferase (ALT, U/L)	138	46	76	157
Platelet count	194	/	/	233
APRI score	3.4	/	/	0.9
NAFLD fibrosis score (FIB-4)	1.08	/	/	0.32
Oral antidiabetic drugs (OADs)	Acarbose 50mg three times daily, metformin 0.5g twice daily	Acarbose 50mg three times daily, metformin 0.5g twice daily, ertugliflozin 5mg once daily	Acarbose 50mg three times daily, metformin 0.5g twice daily, ertugliflozin 5mg once daily	Acarbose 50mg three times daily, metformin 0.5g twice daily, ertugliflozin 5mg once daily
Lipid-lowering drugs	No medication	No medication	No medication	No medication
Other	Bicyclol tablets 50mg, twice daily	Bicyclol tablets 50mg, once daily		

Both patients had type 2 diabetes mellitus (T2DM) and metabolic dysfunction−associated steatotic liver disease (MASLD). Neither patient had hypogonadism or hypothyroidism. HOMA−IR, homeostatic model assessment of insulin resistance; HOMA−β, homeostatic model assessment of β−cell function; eGFR, estimated glomerular filtration rate; APRI, AST−to−platelet ratio index; FIB−4, fibrosis−4 index; OADs, oral antidiabetic drugs. Data are presented as raw values; “/” indicates that the variable was not measured at that time point.

Baseline medications before the addition of ertugliflozin included acarbose 50 mg three times daily, metformin 0.5 g twice daily, and bicyclol tablets 50 mg twice daily for abnormal liver function. Due to poor glycemic control, ertugliflozin 5 mg once daily was added (**Table 2**).

## Results

Both patients received sequential add-on therapy with ertugliflozin 5 mg once daily in addition to their existing antidiabetic regimens.

**Figure 1:**
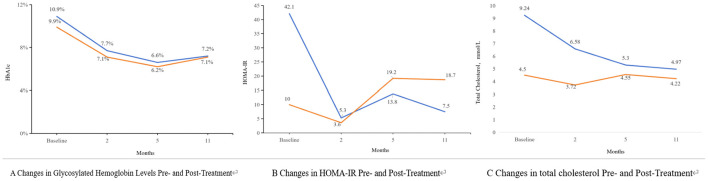
Changes in **(A)** glycosylated hemoglobin, **(B)** HOMA-IR, and **(C)** total cholesterol levels before and after treatment.

**Figure 2:**
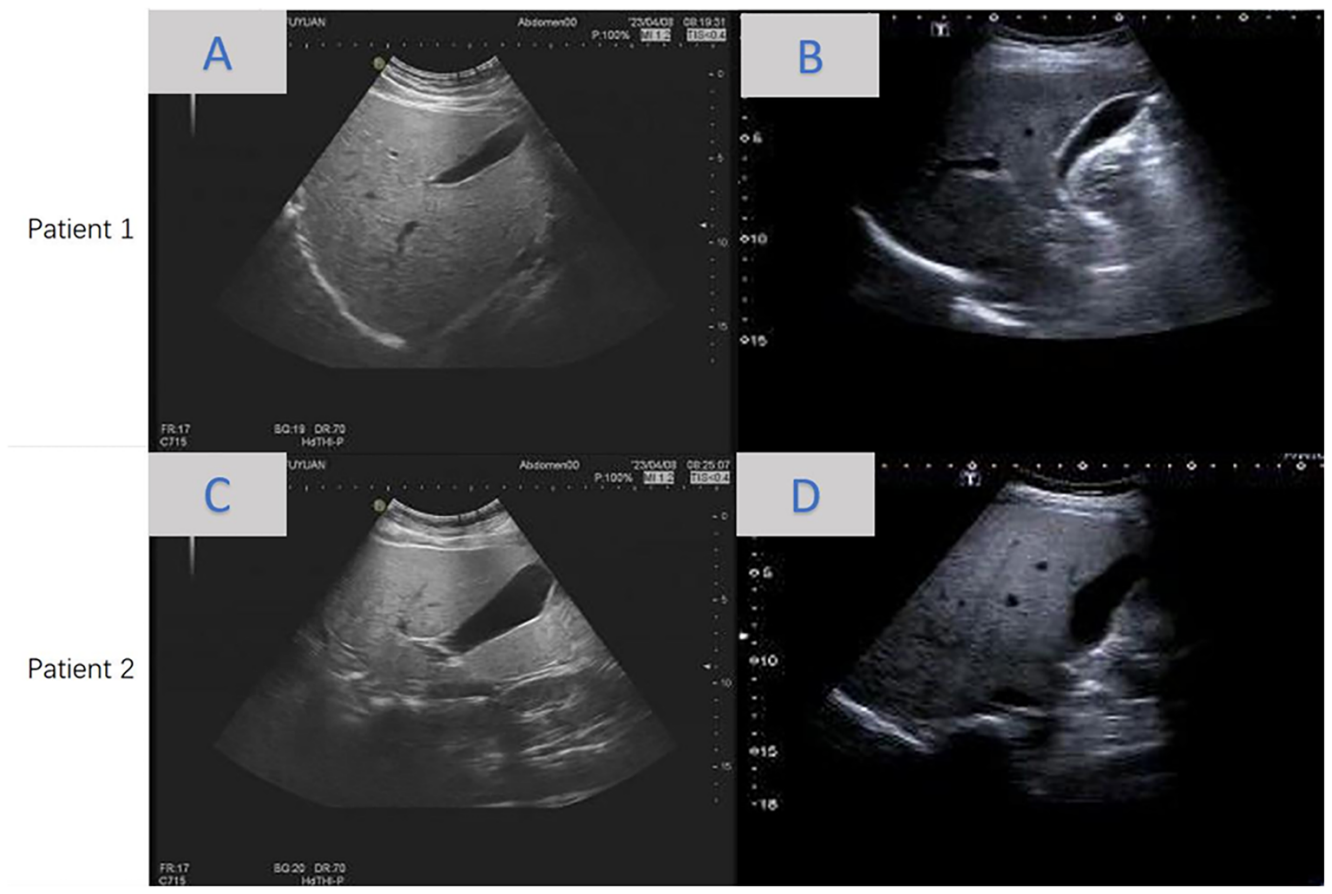
Abdominal ultrasound images of Patient 1 **(A, B)** and Patient 2 **(C, D)** at baseline **(A, C)** and after 11 months of treatment **(B, D)**.

Patient 1: After 11 months of ertugliflozin treatment, multiple parameters improved. Baseline HbA1c was 10.9%, decreasing to 7.7% at 2 months, further to 6.6% at 5 months, and maintained at 7.2% at 11 months (**Figure 1A**), indicating effective and stable glycemic control. HOMA-IR decreased from a baseline of 42.1 to 5.3 (2 months), 13.8 (5 months), and 7.59 (11 months) (**Figure 1B**), indicating significant improvement in insulin resistance. Regarding lipidmetabolism, total cholesterol decreased continuously from a baseline of 9.24 mmol/L to 6.58 mmol/L (2 months), 5.3 mmol/L (5 months), and 4.97 mmol/L (11 months) (**Figure 1C**). Additionally, after 5 months, the uric acid level decreased from a baseline of 711 μmol/L to 635 μmol/L. Other parameter changes are shown in **Table 1**. At baseline, abdominal ultrasound showed mild fatty liver in both patients. After 11 months of treatment, repeat abdominal ultrasound still revealed fatty liver, with no obvious sonographic signs of liver fibrosis or cirrhosis (**Figures 2A, B**). Liver and kidney function remained normal during treatment, with no adverse events observed.

Patient 2: After ertugliflozin treatment, there were significant metabolic improvements, with HbA1c levels decreasing from a baseline of 9.9% to 7.1% at 2 months, 6.2% at 5 months, and stabilizing at 7.1% at 11 months (**Figure 1A**). This indicates effective and stable glycemic control, consistent with findings from the VERTIS-CV study, which involved a large cohort of patients with type 2 diabetes and cardiovascular disease. HOMA-IR decreased from a baseline of 10 to 3.6 (2 months), increased to 19.2 (5 months), and decreased again to 18.7 (11 months) (see **Figure 1B**), suggesting a marked early improvement in insulin resistance followed by partial rebound during follow-up. Regarding lipid metabolism, total cholesterol decreased from a baseline of 4.5 mmol/L to 3.72 mmol/L (2 months), 4.55 mmol/L (5 months), and 4.22 mmol/L (11 months) (see **Figure 1C**). Other parameter changes are shown in **Table 2**. At baseline, abdominal ultrasound showed mild fatty liver in both patients. After 11 months of treatment, repeat abdominal ultrasound still revealed fatty liver, with no obvious sonographic signs of liver fibrosis or cirrhosis (**Figures 2C, D**). Renal function remained normal during treatment, and pre-existing abnormal liver function (associated with fatty liver and fibrosis) did not worsen during the 11-month follow-up. No adverse events were observed.

## Discussion

Insulin resistance in AS is directly caused by mutations in the ALMS1 gene, making it congenital and primary. ALMS1 mutations cause ciliary dysfunction, leading to systemic disruptions in cell signaling and metabolic abnormalities, resulting in primary adipose tissue dysfunction. ALMS1 protein deficiency impairs normal adipocyte differentiation and expansion, severely compromising their ability to store lipids (5). This means that patients cannot safely store energy in adipose tissue, even with normal intake, leading to severe ectopic lipid deposition and forcing large amounts of fat into non-adipose organs, such as the liver, skeletal muscle, pancreas, and heart (6). Consequently, patients with AS exhibit extremely severe insulin resistance (7, 8), hypertriglyceridemia, and fatty liver from early childhood, far exceeding those observed in common obesity. To counter severe insulin resistance, pancreatic β-cells compensate by secreting large amounts of insulin, leading to significant hyperinsulinemia. The liver, a primary site of ectopic deposition, develops severe hepatic steatosis (metabolic dysfunction-associated steatotic liver disease, MASLD) early in the disease course, rapidly progressing to steatohepatitis and fibrosis (9). Hepatic insulin resistance manifests as unsuppressed hepatic glucose output, with persistent glucose overproduction even during fasting, exacerbating hyperglycemia. Similar to type 2 diabetes (T2DM), long-term compensatory hyperinsulinemia eventually leads to β-cell exhaustion (10). However, due to the earlier onset and greater severity of insulin resistance in AS, β-cell functional decline may occur earlier and more rapidly (11).

Given that the mechanism of action of SGLT2 inhibitors is independent of the insulin signaling pathway and directly reduces blood glucose and body weight via urinary glucose excretion, they may offer a unique therapeutic approach for primary insulin resistance diseases such as AS. Large-scale studies evaluating SGLT2 inhibitor use in AS are lacking. Previous case reports indicate good efficacy with other SGLT2 inhibitors (e.g., dapagliflozin and empagliflozin) in patients with AS (3). However, the use of ertugliflozin has not been reported. Our 11-month follow-up of two patients with AS demonstrated significant efficacy of ertugliflozin in glycemic control, lipid metabolism regulation, and insulin sensitivity improvement, consistent with previously published AS case reports.

In this study, two patients with AS treated with the SGLT2 inhibitor ertugliflozin exhibited consistent and clinically relevant reductions in fasting and postprandial glucose concentrations. Effects on lipid metabolism were most pronounced for total cholesterol, particularly in Patient 1, who presented with severe baseline hypercholesterolemia, in whom a sustained reduction of 38% was observed over 11 months. However, as Patient 1 received concomitant bezafibrate therapy throughout the study period, the marked decline in triglycerides (−42% from baseline) cannot be ascribed exclusively to ertugliflozin; rather, it likely reflects a pharmacodynamic synergy between the two agents. Regarding insulin sensitivity, both patients demonstrated an early improvement, evidenced by a reduction in HOMA-IR at the 2-month assessment. Notably, however, Patient 2’s HOMA-IR increased to 18.7 by month 11, indicating attenuation of the initial benefit, potentially attributable to progressive β-cell dysfunction and pubertal insulin resistance. Collectively, these findings suggest a transient, context-dependent enhancement of insulin sensitivity with ertugliflozin in AS. However, given the descriptive nature and limited sample size (n = 2) of this report, no causal inference regarding insulin sensitization can be robustly supported.

First, regarding glycemic control, ertugliflozin was highly effective, and both patients’ fasting blood glucose and HbA1c levels decreased substantially by 2 months. Patient 1 (the elder brother) had a 29.3% reduction in HbA1c (from 10.9% to 7.7%), and Patient 2 (the younger brother) had a 28.3% reduction (from 9.9% to 7.1%). Insulin degludec (20 U/day), which Patient1 was receiving at baseline, was gradually reduced and discontinued one month after adding ertugliflozin. During the 11-month follow-up, both patients maintained HbA1c levels between 6.2% and 7.2%, without ketoacidosis or hypoglycemic events. This indicates that ertugliflozin, an SGLT2 inhibitor, has demonstrated significant glucose-lowering effects and sustained, stable glycemic control in patients with type 2 diabetes, as evidenced by clinical trials showing reductions in HbA1c levels and cardiovascular risks, while maintaining a favorable safety profile. This significant effect is likely attributable to the unique insulin-independent mechanism of SGLT2 inhibitors, which inhibit renal glucose reabsorption and promote glucosuria (12), offering an effective treatment strategy that bypasses the insulin signaling pathway for AS patients with severe insulin resistance.

Second, ertugliflozin demonstrated regulatory effects on lipid metabolism. After 2 months, cholesterol levels decreased substantially in both patients. Patient 1’s cholesterol decreased by 46.2% (from 9.24 mmol/L to 4.97 mmol/L), and Patient 2’s cholesterol decreased by 17.3% (from 4.5 mmol/L to 3.72 mmol/L) at 2 months, remaining relatively stable thereafter. No other cholesterol-lowering medications were used concurrently throughout the treatment period, suggesting that the improvement in cholesterol levels may be attributable to ertugliflozin treatment (13). This finding is significant, indicating that ertugliflozin may help reduce long-term cardiovascular risk in patients with AS (14). Patient 1’s triglyceride levels decreased continuously from a substantial high value of 20.35 mg/dL to 4.61 mg/dL. However, as Patient 1 consistently underwent treatment with a fibrate (bezafibrate, a well-known potent triglyceride-lowering drug with a different and more direct mechanism [PPAR-α activation] than SGLT2 inhibitors) throughout the observation period, the significant triglyceride-lowering effect observed is likely a synergistic result of ertugliflozin and bezafibrate, making it difficult to accurately assess the independent effect of the SGLT2 inhibitor on triglyceride reduction. Furthermore, Patient 1’s uric acid level decreased from 711 μmol/L to 635 μmol/L, suggesting a potential additional benefit of ertugliflozin in lowering uric acid, possibly related to diuresis promoting uric acid excretion and improved insulin resistance.

Both patients showed improvement in insulin sensitivity after ertugliflozin treatment, most notably at the 2-month mark, with significant decreases in HOMA-IR. Patient 1’s HOMA-IR decreased by 87.4% (from 42.1 to 5.3), and Patient 2’s decreased by 64% (from 10 to 3.6). This early effect is likely attributable to the fact that ertugliflozin, as a selective SGLT2 inhibitor, inhibits glucose and sodium reabsorption in the renal proximal tubule, increases urinary glucose excretion, and thereby produces osmotic diuresis (15, 16). This class of drugs does not directly act on insulin receptors or insulin signaling pathways. The observed improvement in insulin resistance is largely an indirect consequence of glucosuria and its downstream metabolic and hemodynamic changes (17). Sustained urinary glucose loss creates a negative energy balance, reduces body weight and visceral fat accumulation, and alleviates glucotoxicity, thereby improving peripheral and hepatic insulin sensitivity (18). Meanwhile, osmotic diuresis mediated by glucosuria reduces extracellular fluid volume and lowers blood pressure, improves vascular function and tissue perfusion, and may further optimize insulin sensitivity (14, 19). Therefore, the diuretic effect of ertugliflozin is a direct osmotic consequence of increased urinary glucose excretion, whereas its improvement of insulin resistance is a multifactorial adaptive result driven by caloric loss, weight reduction, relief of glucotoxicity, and reduced volume load rather than a direct effect of diuresis itself (16–20). Regarding β-cell function, HOMA-β changes showed different patterns. Patient 1’s β-cell function showed an overall downward trend post-treatment, from 619.8 at baseline to 250.3 at 11 months, consistent with improved HOMA-IR and overall lower blood glucose levels, possibly reflecting reduced β-cell burden. In contrast, Patient 2’s HOMA-β showed significant fluctuation and an overall increasing trend, decreasing from 291.4 at baseline to 161.2 at 2 months (the initial decrease possibly reflects reduced burden), followed by an increase to 688.7 at 11 months. Combined with the rebound in HOMA-IR, the later increase in HOMA-β is more likely a compensatory hypersecretory state under persistent insulin resistance. Considering this patient’s obesity and severe primary tissue dysfunction and insulin resistance caused by ALMS1 mutations, which is characteristic of Alström syndrome, ertugliflozin alone may have been insufficient to continuously counteract the underlying disease process over the long term. Over time, disease-inherent factors and puberty-associated hormonal changes that exacerbated insulin resistance may have attenuated its efficacy (8). A case report detailed a significant enhancement in the insulin sensitivity index in a young male patient with AS carrying novel compound heterozygous ALMS1 mutations following treatment with Dapagliflozin, as evidenced by a hyperinsulinemic-euglycemic clamp assessment. Studies suggest that SGLT2 inhibitors, such as dapagliflozin and empagliflozin, may help manage severe insulin resistance in AS, which is consistent with our research findings.

**Figure 3:**
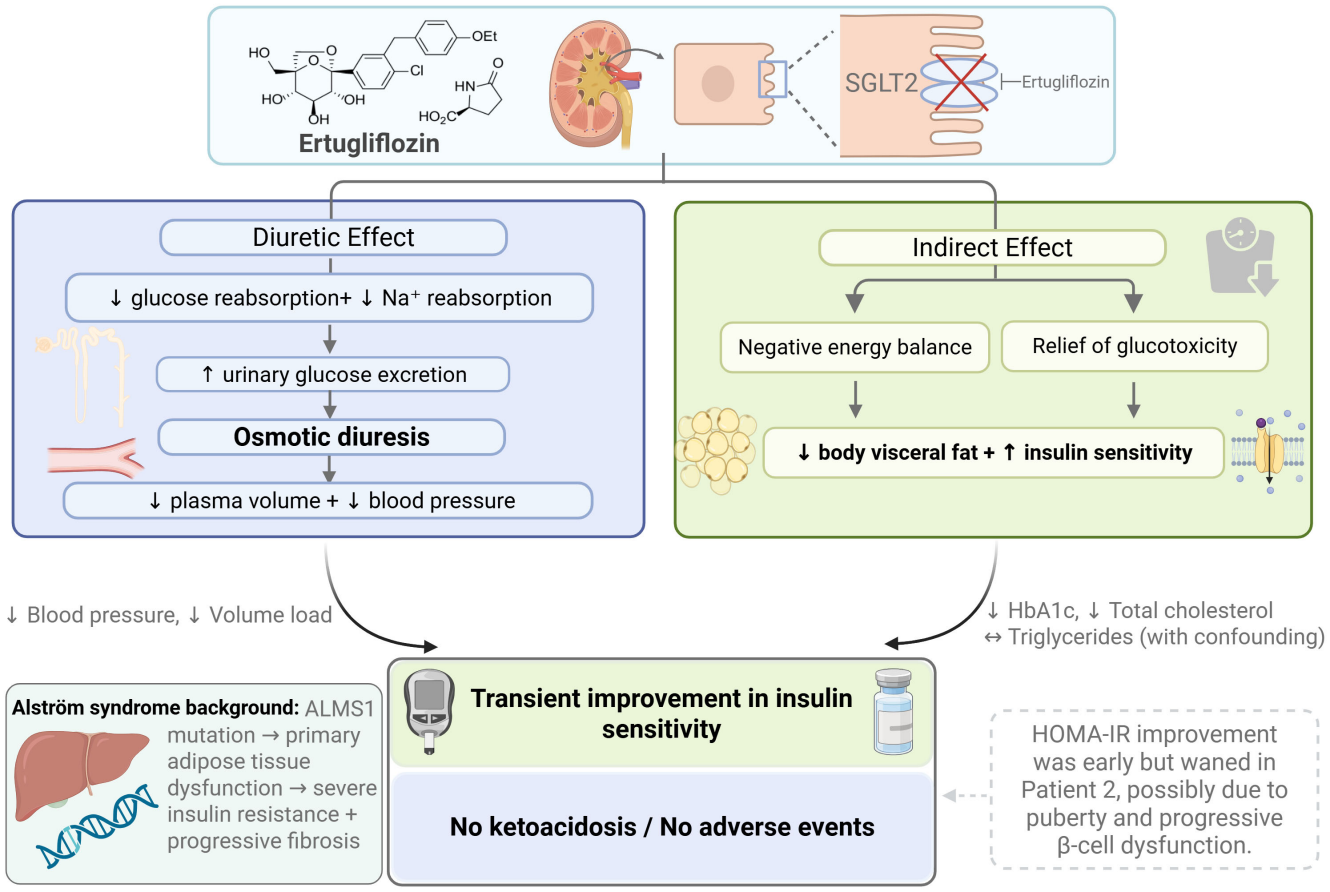
Schematic diagram illustrating the proposed mechanisms of ertugliflozin in patients with Alström syndrome. Ertugliflozin inhibits SGLT2 in the renal proximal tubule, leading to osmotic diuresis (↓ plasma volume and blood pressure) and metabolic benefits (↓ body weight/visceral fat, ↓ glucotoxicity, and ↑ insulin sensitivity). No ketoacidosis or serious adverse events occurred. Dashed lines indicate pathways with limited or confounding evidence. ALMS1, Alström syndrome 1; SGLT2, sodium-glucose cotransporter 2.

The above mechanistic cascade is illustrated in **Figure 3**, which provides a visual summary of the key pathways discussed. Beyond efficacy, however, the safety profile of SGLT2 inhibitors, particularly the risk of ketoacidosis, deserves careful attention.

Ketoacidosis is a known serious adverse reaction associated with SGLT2 inhibitors and has been reported in clinical trials of ertugliflozin (21). In the present study, neither of the two patients with AS developed ketoacidosis during the 11-month follow-up period. Previous literature has reported that the occurrence of ketoacidosis is closely associated with very-low-carbohydrate diets or prolonged fasting. The two patients in this study followed a traditional northern Chinese diet, characterized by wheat-based staples as the core carbohydrate source, supplemented with vegetables, meat, and other side dishes. Since the two patients first presented to our center in 2022, we have recommended a standard diabetic diet to the patients and their guardians based on the Chinese Guidelines for the Prevention and Treatment of Type 2 Diabetes, along with strict lifestyle intervention. This dietary regimen emphasizes controlling total caloric intake, optimizing carbohydrate composition (increasing whole grains and dietary fiber while reducing refined sugars and high-glycemic-index foods), balancing protein and fat intake, maintaining regular meal timing and portions, and making appropriate adjustments according to changes in the patients’ height, weight, and daily physical activity. This dietary pattern does not constitute a very-low-carbohydrate or ketogenic diet. On the other hand, although SGLT2 inhibitors have a ketogenic effect (21), the elevation in ketone bodies typically remains within a non-pathological range when glycemic control is relatively adequate and insulin deficiency is not severe. Furthermore, in recent years, the role of gut microbiota in metabolic regulation and the development of ketoacidosis has gained increasing attention. Studies have shown that the abundances of genera such as Prevotella and Selenomonas in the oral cavity of patients with Alström syndrome are elevated. These genera are closely associated with type 2 diabetes, obesity, and insulin resistance, and their increase is often related to a high-sugar diet. Meanwhile, the abundance of Lactobacillus in the oral cavity of these patients is significantly reduced. Lactobacillus has been shown to improve insulin sensitivity, reduce body weight, and exert anti-inflammatory effects; therefore, its reduction may diminish these protective effects and increase the risk of obesity, type 2 diabetes, and diabetic ketoacidosis (DKA) (22). This suggests that Alström syndrome may exacerbate systemic inflammation and metabolic instability by promoting the overgrowth of specific microbial taxa. Another study indicated that the gut commensal Akkermansia muciniphila is a protective factor against DKA, and its abundance is significantly negatively correlated with the risk of DKA and delayed diagnosis (23). Patients with AS may also exhibit microbiota dysbiosis associated with ALMS1 gene mutations, which could have complex effects on ketone metabolism. Future studies are warranted to further investigate the gut microbiota characteristics in patients with AS and their impact on the response to SGLT2 inhibitor therapy. Such investigations may provide new insights for personalized treatment in this population.

During the 11-month follow-up, neither patient experienced adverse events such as hypoglycemia, ketoacidosis, genitourinary infections, dehydration, or hypotension, which is consistent with the safety profile of ertugliflozin observed in the VERTIS CV study (15). Patient 1 showed no significant abnormalities in liver or kidney function. The estimated glomerular filtration rate (eGFR) decreased slightly from 117 mL/(min·1.73m²) to 88 mL/(min·1.73m²), while urine microalbumin remained within the normal range throughout follow-up, indicating minimal renal/hepatic impact from ertugliflozin. Patient 2 showed no significant renal abnormalities. His eGFR increased from 251 mL/(min·1.73m²) to 269 mL/(min·1.73m²), and urine microalbumin remained normal. However, Patient 2’s alanine aminotransferase (ALT) levels fluctuated during treatment. Given his baseline abnormal liver function, these fluctuations were considered related to his obesity, fatty liver, and liver fibrosis, suggesting that the drug-mediated improvement of liver inflammation may be limited against the backdrop of AS’s inherent progressive liver damage (9). Therefore, long-term monitoring remains warranted. In addition, during long-term follow-up after the completion of treatment, we performed non-invasive liver fibrosis assessments in both patients. At 24 months after treatment completion (approximately 35 months after the first dose), patient 1 underwent transient elastography, which showed a liver stiffness value of 9.2 kPa, indicating mild-to-moderate cirrhosis (stage F2–F3). Patient 2 had a value of 7.3 kPa, with no apparent progression compared with baseline. These long-term follow-up data suggest that, despite the clear metabolic improvements observed during ertugliflozin treatment, the progressive liver fibrosis driven by ALMS1 mutations in Alström syndrome may continue to progress slowly. Both patients remain in our long-term follow-up cohort, and have now been monitored for four years since their initial presentation. Throughout the entire follow-up period, no elevated blood pressure has been observed nor have any clinical symptoms potentially related to blood pressure abnormalities, such as dizziness, headache, or palpitations, been reported. Although ertugliflozin demonstrated a favorable safety profile in these two patients, the known potential adverse effects of SGLT2 inhibitors continue to warrant attention.

This case report has inherent limitations, including a small sample size, lack of a control group, insufficient follow-up duration for assessing long-term outcomes, and concurrent use of metformin and acarbose in both patients (and lipid-lowering drugs in Patient 1), which constitute significant confounding factors affecting the assessment of SGLT2 inhibitor-specific benefits on lipid metabolism. In this study, no invasive histopathological examinations were performed on the patients, and pre- and post-treatment liver and kidney tissue-level data were unavailable. These limitations compromise a comprehensive evaluation of drug safety.

In conclusion, this study suggests that ertugliflozin, as a member of the SGLT2 inhibitor family, may provide metabolic benefits in patients with Alström syndrome, particularly a definite glucose-lowering effect and a possible reduction in total cholesterol. The possible improvement in insulin sensitivity and triglyceride levels requires further investigation with larger sample sizes and longer follow-up, ideally with appropriate controls. Future larger, long-term studies are needed to confirm its efficacy and safety and explore its therapeutic role in this complex disorder.

AS involves multiple systems, requiring multidisciplinary management, including endocrinology, nephrology, ophthalmology, ENT, and cardiology. Diabetes and its comorbidities require a multifaceted, combined approach that integrates plant-based diets, nutraceuticals, and conventional pharmacotherapy. These components are complementary and can more effectively control blood glucose, improve metabolic disturbances, and reduce cardiovascular risk (24, 25). For rare and complex metabolic diseases such as AS, such a multifaceted approach is of great value. Although pharmacotherapy can provide clear metabolic benefits, individualized nutritional support and long-term multidisciplinary management remain essential. Future studies should further explore comprehensive intervention strategies suitable for patients with AS, including optimized dietary modifications, the use of nutraceuticals, and optimized drug combinations. SGLT2 inhibitors are integral to a comprehensive management strategy for diabetes, offering not only symptomatic relief but also additional benefits such as cardiovascular and renal protection and potential cognitive health advantages. In this study, two siblings with AS carrying the same compound heterozygous ALMS1 mutations (c.1894C>T and c.9148_9149delCT) both demonstrated multifaceted metabolic improvements after treatment with ertugliflozin (5 mg once daily), reaffirming the potential of this drug class for managing metabolic complications in AS.

## Data Availability

The original contributions presented in the study are included in the article/supplementary material. Further inquiries can be directed to the corresponding authors.
